# A Homeobox Transcription Factor UvHOX2 Regulates Chlamydospore Formation, Conidiogenesis, and Pathogenicity in *Ustilaginoidea virens*

**DOI:** 10.3389/fmicb.2019.01071

**Published:** 2019-06-20

**Authors:** Junjie Yu, Mina Yu, Tianqiao Song, Huijuan Cao, Xiayan Pan, Mingli Yong, Zhongqiang Qi, Yan Du, Rongsheng Zhang, Xiaole Yin, Yongfeng Liu

**Affiliations:** ^1^Institute of Plant Protection, Jiangsu Academy of Agricultural Sciences, Nanjing, China; ^2^School of the Environment and Safety Engineering, Jiangsu University, Zhengjiang, China

**Keywords:** *Villosiclava virens*, chlamydospore, conidiation, pathogenicity, homeobox transcription factor

## Abstract

Rice false smut fungus (teleomorph: *Villosiclava virens*; anamorph: *Ustilaginoidea virens*) can generate chlamydospores and survive winter under field conditions. The chlamydospore is considered as an important infection source of the disease. However, little is known about the regulatory mechanism of the chlamydospore production. In this study, we identified a defective homeobox transcription factor (designated as UvHOX2) gene in a *U. virens* random insertional mutant B-766 that could not form chlamydospores. To confirm the regulatory function of UvHOX2, an *Agrobacterium tumefaciens* mediated transformation- and CRISPR/Cas9- based targeted gene replacement method was developed. The *UvHox2* deletion mutants completely failed to produce chlamydospores, showed reduced conidia production and decreased virulence, and was hyper-sensitive to oxidative, osmotic, and cell wall stresses. We confirmed that UvHOX2 is located in the nuclei of *U. virens*, and the expression of *UvHox2* was the strongest during the early stage of chlamydospore and conidium formation. Global transcription pattern of UvHOX2 was also determined by RNA-seq in this study, and several genes that might be down-stream of UvHOX2 regulation were identified. The results will better our understanding of the molecular mechanism of chlamydospore formation in *U. virens* as a model fungus.

## Introduction

The heterothallic ascomycetous fungus *Ustilaginoidea virens* (Cooke) Takah (Teleomorph: *Villosiclava virens*) is the causal agent of rice false smut (RFS), which has become an emerging disease in China and most rice-growing areas in the world (Guo et al., 2012; Nessa et al., 2015; Yu J.J. et al., 2015). The fungus infects stamen filaments of rice at the booting stage, hijacks rice nutrients, and transforms kernels into false smut balls containing a large number of yellow or dark green-pigmented chlamydospores (Hu et al., 2014; Meng et al., 2015; Song et al., 2016; Zheng et al., 2016). Occasionally, sclerotia could form on the surface of false smut balls in late autumn when the temperature falls (Yu et al., 2016; Yong et al., 2018). Previous studies have revealed that chlamydospores could survive in nature and play an important role in the epidemiology of RFS disease between seasons (Fan et al., 2016).

In the human pathogenic yeast *Candida albicans*, several genes were found to be involved in chlamydospore formation, including homeobox transcription factor (TF) gene *grf10* (Ghosh et al., 2015), phosphate mannose synthase encoding genes (Juchimiuk et al., 2015), mitogen-activated protein kinase gene *hog1* (Eisman et al., 2006), gene encoding dolichol phosphate mannose synthase (Juchimiuk et al., 2015), chromatin remodeling complex gene *isw2* (Nobile et al., 2003; Navarathna et al., 2016), *MDS3, RIM101, RIM13, SCH9*, and *SUV3* (Nobile et al., 2003). Meanwhile, very few genes in filamentous fungi were found relative to chlamydospore formation, the limited examples include VELVET gene *vel1* in biocontrol fungus *Trichoderma virens* (Mukherjee and Kenerley, 2010); and a group of genes in *Clonostachys rosea* identified from a study using RNA-seq (Sun et al., 2018). So far, the molecular mechanisms of chlamydospore formation remain elusive in filamentous fungi.

Homeobox genes encode a group of TFs, which contain a conserved homeobox domain and bind to specific DNA sequences (Gehring, 1987). In eukaryotic cells, these homeobox TFs play an important role in regulation of cell differential and development (Liu et al., 2010; Antal et al., 2012). The first reported homeobox gene in filamentous ascomycetes is *pah1* in *Podospora anserine* (Arnaise et al., 2001). *Pah1* deletion mutant showed increased production of microconidia and reduced growth rate of mycelia. In model fungus *Neurospora crassa*, three homeobox genes were characterized (Colot et al., 2006). Specifically, deletion of *kal-1*(*pah1* homolog)led to defects in mycelia growth and conidiation; *bek-1* was found to be essential for perithecial development whereas the third homeobox gene (Genbank accession number: NCU03070) was not described.

In recent years, several homeobox genes were systematically studied in filamentous fungi *Porthe oryzea* and *Podospora anserine*, and the results confirmed that these homeobox genes play a regulatory role in conidium and fruiting body development, as well as host infection (Kim et al., 2009; Coppin et al., 2012).

In this study, we identified a chlamydospore formation defect *U. virens* mutant B-766 from a random insertional mutant library that was constructed previously (Yu M.N. et al., 2015). A homeobox gene (annotated as *UvHox2*) was confirmed to be involved in the regulation of chlamydospore formation and pathogenicity in *U. virens*. A CRISPR/Cas9 system based on *Agrobacterium tumefaciens* mediated transformation (ATMT) was developed for targeted gene deletion. Moreover, comparative transcriptional analysis of *UvHox2* deletion mutant and a wild-type strain was performed in this study. Taken together, the findings from this work will help us understand the regulatory mechanism of chlamydospore formation better.

## Materials and Methods

### Strains, Rice Variety, Plasmids, and Nucleotide Acids Manipulation

A virulent wild-type *U. virens* strain P-1 was used as starting strain in this study. A rice variety susceptible to *U. virens*, Liangyoupeijiu, was used in the inoculation experiments. The plasmid pCas9-tRp-gRNA was kindly provided by Dr. Jingrong Xu at Northwest A&F University (Liang et al., 2018). *A. tumefaciens* strain AGL-1, plasmid pmCherry-hph, pCambia-1300, pBHt2, pKHt, and pCN3EXPS were from our lab. Southern blot and thermal asymmetric interlaced PCR (TAIL-PCR) were performed as described previously (Yu M.N. et al., 2015).

### Phenotypic Analysis of *U. virens* Strains/Mutants

The *U. virens* wild-type strain P-1 was routinely cultured on a potato sucrose agar medium (PSA) at 28°C for 10–15 days (Zheng et al., 2017). The transformants of P-1 were cultured on the PSA amended with 100 μg/ml hygromycin and/or 600 μg/ml geneticin 418 (G418). We used YT medium and broth to test mycelial growth rate and conidiation ability of *U. virens*, respectively (Tanaka et al., 2011). To determine the chlamydospore formation and the pathogenicity of *U. virens* strains, we inoculated rice following the method described previously (Zheng et al., 2017). Fifteen spikes were inoculated for each strain, and the number of false smut balls was counted 25 days after the inoculation. The chlamydospore formation structures on the surface of false smut balls were observed by scanning electron microscope (SEM). To stimulate chlamydospore formation in *U. vires*, mycelia dishes cut from the edge of fresh colonies were put on PSA medium. The cultures were incubated at 28°C under diffuse light for 2–3 months.

*Ustilaginoidea virens* strains were cultured on PSA medium to determine the growth rate. YT medium amended with 0.05% H_2_O_2_, 0.4 mol/l NaCl, 0.03% SDS, and 100 mg/l congo red were used to test sensitivity of stains to abiotic stresses. The cultures were incubated at 28°C for 15 days in darkness, and then the growing diameter was measured and the morphology of the colonies were characterized. Four duplicates were performed for each treatment. The sporulation capacity of the strains was determined as follows. Ten pieces of fresh mycelial dishes from each treatment were cultured in a 250 ml conical flask containing 100 ml YT liquid medium. The conical flasks were incubated at 28°C, 150 r/min for 6 days, and then the thin-wall conidia were counted with a blood cell counting chamber. To observe conidium generation structures, strains were cultured on minimal media (MM) (Gupta and Chattoo, 2008) for 10 days.

### Generation of ATMT Binary Vector for Gene Deletion With CRISPR/Cas9

#### Generation of Gene Deletion Vector With CRISPR/Cas9

We constructed CRISPR-guideRNA(gRNA) cassettes from pCrispr-UvtR and gene replacement cassettes [upstream flank (UF)-hygromycin resistant gene(Hyg^+^)-downstream flank (DF)] of *UvHox2* into two T-DNA regions of binary vector pCccd-dTN3, respectively. The details of vectors construction were described in Supplementary Figure S1.

#### Generation of Gene Deletion Mutants

*Agrobacterium tumefaciens* mediated transformation was performed as described previously (Yu M.N. et al., 2015). The *A. tumefaciens* strains AGL-1 containing pdTN3-HX2-Cas9I or pdTN3-HX2-Cas9II was employed in transformation of *U. virens* wild-type strain P-1. The *U. virens* P-1 and *A. tumefaciens* AGL-1 were co-cultured on nitrocellulose membrane for 3 days and then transferred onto 2% TB3 [0.3% yeast extract, 0.3% casamino acid, 1% glucose, 2% sucrose (w/v)]. To make a selective medium, 400 μg/ml cefotaxime and 150 μg/ml timentin were added into 2% TB3 medium to inhibit the growth of *A. tumefaciens*, and 100 μg/ml hygromycin and 600 μg/ml G418 were added into 2% TB3 medium to select transformants containing both cassettes of UF-HYG^+^-DF and CRISPR/Cas9-gRNA, respectively. The *UvHox2* deletion mutants were screened and verified by PCR with primers P19∼P26 listed in Supplementary Table S1.

#### Generation of UvHox2-eGFP Constructs

The open reading frame (ORF) of *UvHox2* was amplified from cDNA that was generated through reverse transcription of total RNA using the primer pair P27–P28 (Supplementary Table S1). The enhanced green florescent protein (eGFP) fragment was amplified with primer pair P29–P30 (Supplementary Table S1). UvHox2-eGFP fusion cassette was generated via double-joint PCR and ligated to *Bam*H I-*Eco*R I digested pCN3EXPS to construct UvHox2-eGFP fusion vector pCN3EXPS-HX2-eGFP, in which the UvHox2-eGFP cassette was under the control of glyceraldehyde-3-phosphate dehydrogenase promoter of *Cochliobolus heterostrophus*. Subsequently, the vector was used to transform *U. virens* via ATMT protocol to generate UvHox2-eGFP over-expression mutants.

#### qRT-PCR Assays

Vegetative mycelia were collected from 2-day-old YT cultures that started with 1 × 10^6^ conidia/ml. To stimulate sporulation in *U. virens*, mycelial dishes were cultured in YT broth by shaking for 3 days (initial stage of sporulation) or 7 days (later stage of sporulation). To collect samples undergoing chlamydospores formation, 20 rice spikes were inoculated for each strain/mutant. Rice smut balls at the initial stage [yellowish with intact membrane] and the later-stage [yellowish without membrane] of chlamydospore development were collected as described by Fan et al. (2016). PrimeScript^TM^ RT reagent Kit with gDNA Eraser (Takara) and SYBR^®^ *Premix Ex Taq^TM^* II (Takara) were used to synthesize cDNA and quantitative RT-PCR (qRT-PCR). Relative expression levels of genes were calculated with the 2-ΔΔCt method. The α-tubulin gene was employed as the endogenous reference. Three biological replicates were performed to calculate the mean and the standard deviation.

#### Comparative Transcriptional Analysis of *U. virens*

Total RNA of *U. virens* was extracted employing TRIZOL (Invitrogen). RNA integrity was determined using Bioanalyzer 2100 RNA-6000 Nano Kit (Agilent Technologies). The construction and sequencing of mRNA-seq libraries and preprocessing and mapping of Illumina reads were performed as described previously (Yu et al., 2016). The DESeq software (Anders and Huber, 2012) was used to generate base mean based on FPKM, and to evaluate significant differences in base mean between different samples. Three biological replicates were performed for each strain/mutant.

## Results

### Characterization of Genes Relative to Chlamydospore Formation in Mutant B-766

**FIGURE 1:**
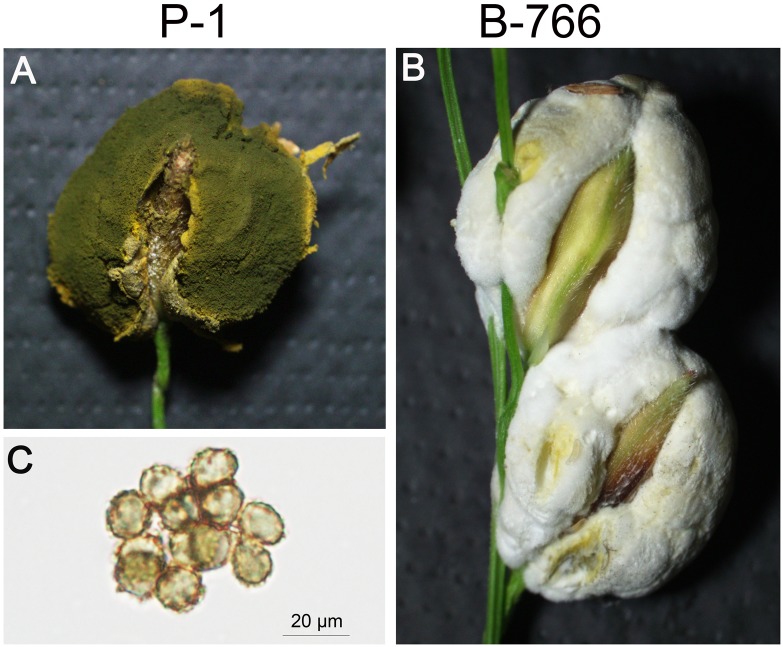
Rice false smut (RFS) balls of wild-type strain P-1 and T-DNA insertional mutant B-766 of *U. virens*. **(A)** RFS balls of wild-type strain P-1. **(B)** RFS balls of mutant B-766. **(C)** The chlamydospores formed on the false balls of wild-type strain P-1.

**FIGURE 2:**
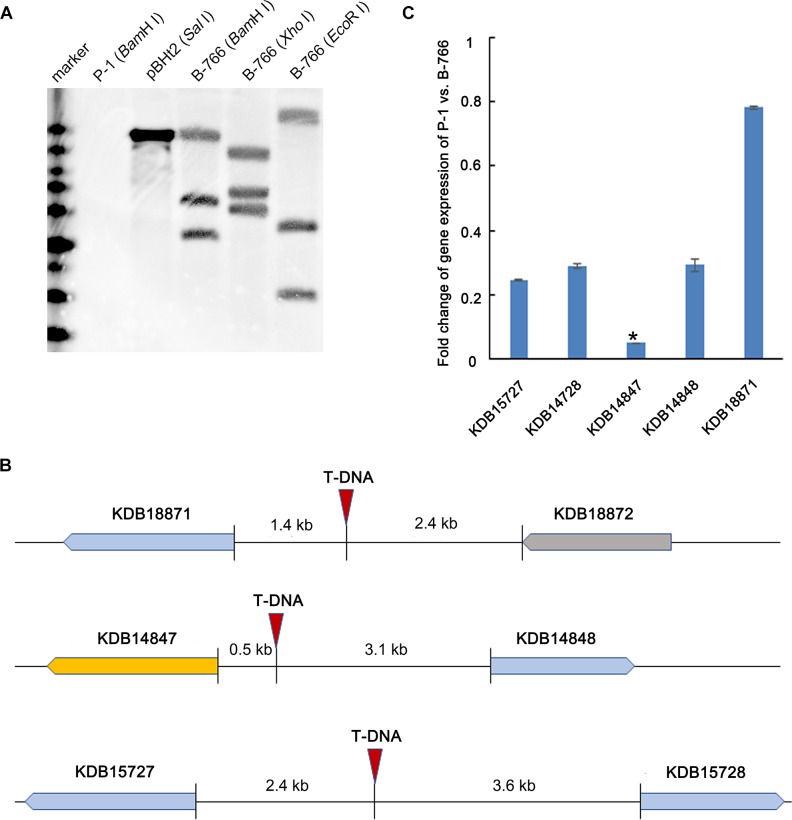
Characterization of T-DNA insertional mutant B-766. **(A)** Copy number analysis of T-DNA in B-766 by southern blot. **(B)** Illustration of insertion sites of T-DNA in B-766. **(C)** Fold change of gene expression of wild-type stain P-1 comparing to mutant B-766. The asterisk indicated that the fold change ofKDB14847 in B-766 comparing to P-1 of was significantly higher than KDB15727, KDB14728, KDB14848, and KDB18871.

In a preliminary study, we identified a T-DNA insertional mutant B-766 of *U. virens*, which failed to form chlamydospores on false smut balls (Figure 1). To determine the copy number of T-DNA inserted in B-766, 1.4 kb hygromycin resistant cassette was employed as a probe in southern blot. The result showed that three copies of T-DNA were detected in mutant B766 (Figure 2A). T-DNA flanking regions were amplified by TAIL-PCR (Yu M.N. et al., 2015). Three copies of T-DNA were inserted into the upstream of ORFs that encode proteins KDB15727 (Genbank accession number), KDB15728, KDB14847, KDB14848, and KDB18871 (Figure 2B). We then performed qRT-PCR to screen genes relative to chlamydospore formation in mutant B-766. The expression of KDB14847 in B-766 comparing to P-1 was reduced in a higher level than other genes that might be infected by T-DNA insertion in mutant B-766 (Figure 2C). Because KDB14847 is homologous to homeobox TF MoHOX2 in *Magnaporthe oryzea*, we designated KDB14847 as UvHOX2.

### Homeobox TFs in *U. virens*

**FIGURE 3:**
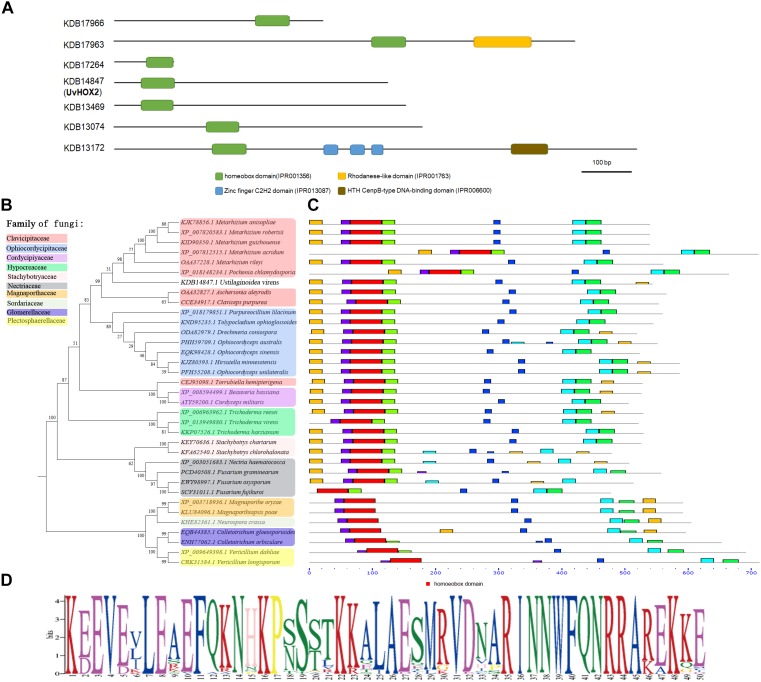
Phylogenetic analysis of homeobox transcription factors (TFs) in *U. virens*. **(A)** Positions of homeobox domain in homeobox TFs in *U. virens.* **(B)** Phylogenetic analysis of homeobox TFs that homologous to UvHOX2 in filamentous fungi. **(C)** Positions of homeobox domain (red) in UvHOX2 homologs. **(D)** Sequences of homeobox domain in filamentous fungi.

In eukaryotic cells, homeobox TFs contain a ∼60 aa long conserved homeodomain that binds to specific DNA sequences and regulates transcription (Coppin et al., 2012). We identified seven homeobox TFs in *U. virens* using the InterPro term (IPR001356) and the NCBI assembly data of *U. virens* genome (accession number: GCA_000687475.1). The sequences of the homeobox TFs were deposited in the genbank with the accession number KDB17966, KDB17963.1, KDB17264, KDB14847, KDB13469, KDB13074, and KDB13172 (Figure 3A). The position of the homeodomain varied in the homeobox TFs in *U. virens*. The homeobox motif located at the N-terminus of KDB14847 (UvHOX2), KDB13469, and KDB13172, in the middle of KDB17963 and KDB13074, and at the C-terminus of KDB17966 (Figure 3A–D). Besides the homeobox domain, KDB17963 possessed a rhodanese-like domain at the C-terminus, KDB13172 harbored three zinc finger C2H2 DNA binding domains in the middle and an HTH CenpB-type DNA binding domain at the C-terminus (Figure 3A).

### Generation of UvHOX2 Deletion Mutants

**FIGURE 4:**
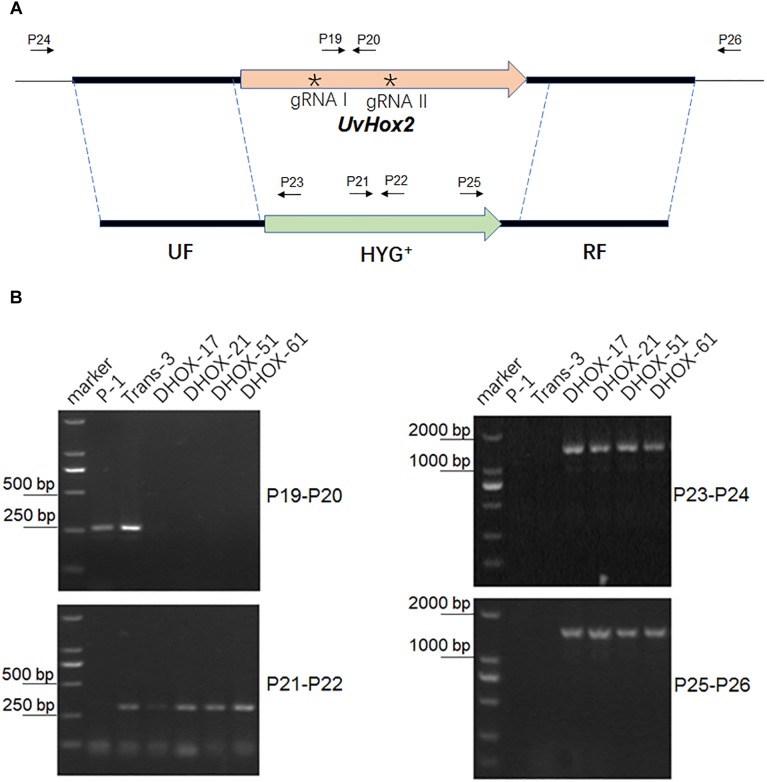
Deletion of the *UvHOX2* gene in *U. virens*. **(A)** Illustration of targeted deletion of *UvHOX2* utilizing CRISPR/Cas9 and homologous replacement. **(B)** PCR analysis of *UvHOX2* deletion mutants DHOX-17, DHOX-21, DHOX-51, and DHOX-61. The wild-type strain P-1 and transformant Trans-3 with ectopic inserted UF-HYG^+^-RF cassette were included as controls. The asterisks indicates the positions of gRNA spacers (gRNA I and gRNA II) designed for CRISPR/Cas9.

To delete *UvHox2* in the *U. virens* wild-type strain P-1, we constructed two binary vectors pdTN3-HX2-Cas9I or pdTN3-HX2-Cas9II containing two T-DNA regions, respectively. The first T-DNA inserted with UF-HYG^+^-RF cassette, and the other one harbors CRIPSR/Cas9-gRNA (gRNA1 or gRNA2) and NEO^+^ cassettes. The ATMT method was used to co-transfer the two T-DNAs into the *U. virens* wild-type strain P-1. The transformants resistant to hygromycin B and G418 were picked from the selective medium after about 7–10 days culture at 28°C in darkness. For pdTN3-HX2-Cas9I, 9 out of 33 transformants were confirmed as *UvHox2* deletion mutants. For pdTN3- HX2-Cas9II, 10 out of 45 transformants were confirmed as *UvHox2* deletion mutants (Figure 4). The homologous gene replacement frequency was 27.3 and 22.2% for pdTN3-HX2-Cas9I and pdTN3-HX2-Cas9II, respectively. Potential off-target sites were checked using protocol described by Liang et al. (2018), and no off-target event was detected in all transformants.

### *UvHox2* Is Essential for Chlamydospore Formation and Contributes to Pathogenicity

**FIGURE 5:**
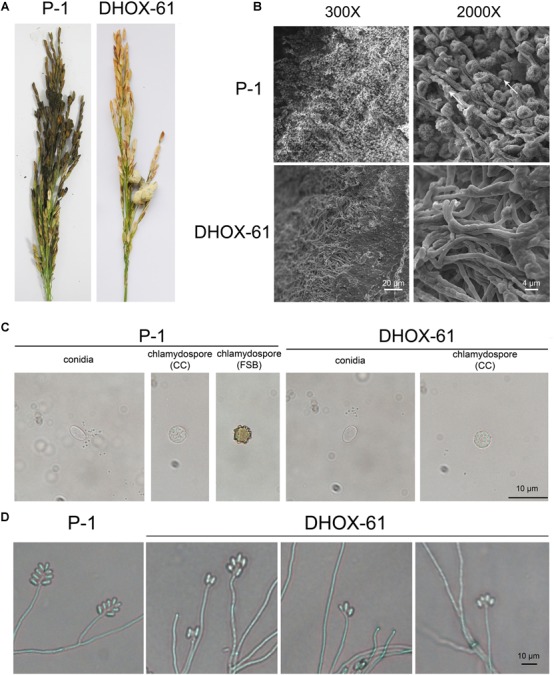
Rice false balls of wild-type strain P-1 and *UvHOX-2* deletion mutant DHOX-61. **(A)** Rice spikes inoculated with P-1 or DHOX-61. **(B)** Chlamydospores on the surface of rice false ball of P-1 or DHOX-61 observed under a scanning electron microscope with 300× and 2000× amplification, respectively. The arrows indicate the short peg-like branches (sterigmata) were observed on the parallel arranged sporangiophore-like mycelia. **(C)** Chlamysopores of P-1 or DHPX-61 converted from conidia (CC) or formed on RFS balls (FSB). **(D)** Sporulation structures of P-1 or DHOX-61.

*UvHox2* deletion mutants DHOX17 and DHOX21 generated by pdTN3-HX2-Cas9I and DHOX51 and DHOX61 generated by pdTN3-HX2-Cas9II were then used to inoculate rice at booting stage on 15 spikes. Disease incidence of RFS was detected 25 days after inoculation. The rice smut balls were observed once again 60 days after innoculation to check the chlamydospore formation. The results showed that the virulence of the *UvHox2* deletion mutants was significantly reduced compared to the wild-type strain P-1. The number of RFS balls on the spikes inoculated with the mutants DHOX-17, DHOX-21, DHOX-51, and DHOX-61 were 3.8 ± 1.6, 4.7 ± 2.6, 4.3 ± 1.6, and 4.3 ± 1.8, respectively; meanwhile, the number of smut balls on spikes inoculated with the wild-type strain P-1 was 21 ± 3.5 (Figure 5A and Table 1). The false smut ball samples were fixed with 2.5% glutaraldehyde in phosphate buffer pH 7.4 and subsequently observed under a SEM. The pictures showed that the chlamydospore formation structure was normal on the surface of false smut balls on the wild-type strain inoculated spikes. Short peg-like branches (sterigmata) were observed on the parallel arranged sporangiophore-like mycelia, and chlamydospores formed on these sterigmata in the wild-type strain P-1. In contrast, no special structure of hypha was observed on the false smut balls of *UvHox2* deletion mutants, and the hyphae were abnormally curved and twined (Figure 5B). To test whether conidia of *U. virens* wild-type strain (WT)/mutants could occasionally be converted into chlamydospores in the absence of water, conidia of *U. virens* WT/mutants were placed on glass slides and kept in the 9 mm-diameter petri dishes. After 10-days of incubation at 28°C in darkness, we found a small portion of conidia wild-type strain P-1 and *UvHox2* deletion mutants could both convert into chlamydospores (Figure 5C). The finding implied that UvHOX2 plays a key regulatory role in formation of chlamydospores rather than conversion from specific cells into chlamydospores.

**Table 1:** Strains and vectors used in the study.

Strain	Description in brief	References
P-1	Wild-type strain of *U. virens*	Zheng et al., 2017
DHOX-17	*UvHOX2* deletion mutant	This study
DHOX-21	*UvHOX2* deletion mutant	This study
DHOX-51	*UvHOX2* deletion mutant	This study
DHOX-61	*UvHOX2* deletion mutant	This study
Trans-3	UF-HYG^+^-RF ectopic insertion mutant	This study
HOX-GFP-2	HOX-GFP fusion expression mutant	This study
Vector		
pmCherry-hph	Harboring mcherry expression- and hygromycin resistant cassettes	Stock of our lab
pCambia-1300	Binary vector for agrobacterium tumefaciens transformation (ATMT)	Cambia
pBHt2	Binary vector for ATMT of filamentous fungi	Mullins et al., 2001
pKHt	Binary vector containing eGFP expression cassette	Mullins et al., 2001
pCN3EXPS	Binary vector for over-express of genes in filamentous fungi	Stock of our lab
AGL-1	Agrobacterium tumefaciens strain	Presented by Dr. Lijun Ma
pCas9-tRp-gRNA	Containing CRISPR/Cas9 cassette and gRNA cassettes controlled by Gln-tRNA	Liang et al., 2018
pCas9-tRPD	Two *Aar* I restriction sites were introduced in the flanking regions of CRISPR/Cas9 and gRNA cassettes	This study
pCas9-tRPDI	pCas9-tRPD inserted with gRNA spacers SP1S-SP1AS	This study
pCas9-tRPDII	pCas9-tRPD inserted with gRNA spacers SP2S-SP2AS	This study
Pcccd	Binary vector containing a ccdB toxic cassette in T-DNA 1 region	This study
pCccd-dT	Binary vector containing two T-DNA regions	This study
pCccd-dTN3	A geneticin 418-resistant cassette added into T-DNA 1 region	This study
pdTN3-HX2-Cas9I	For deletion of *UvHOx2*	This study
pdTN3-HX2-Cas9II	For deletion of *UvHOx2*	This study

### UvHOX2 Regulates Conidiogenesis

The conidial production of *UvHox2* deletion mutants in YT broth was significantly reduced in comparison to that of the wild-type strain P-1 (Table 2). To observe the intact conidial sporulating structures, we cultured these strains (WT/mutants) on MM media. Wild-type strains produced typical conidial sporulating structures, but 86% of the *UvHox2* deletion mutants produced abnormal sporulating structures (Figure 5D). Whereas, the conidia produced by the wild-type strain and the *UvHox2* deletion mutant were the same in appearance.

**Table 2:** Pathogenicity, conidiation, and conidial germination in *UvHOX2* deletion mutants.

Strain	Mycelium growth (mm)	Pathogenicity^a^	Concentration of conidia in YT broth (Log_10_ of spores/ml)	Percentage of abnormal sporulating structures(%)	Percentage of conidial germination(%)
P-1	53.88 ± 2.61 A^b^	21 ± 3.5 A	6.88 ± 0.02 A	19.25 ± 4.66 B	90.56 ± 1.45 A
DHOX-17	44.75 ± 1.92 B	3.8 ± 1.6 B	6.78 ± 0.02 B	79.5 ± 4.72 A	90.71 ± 1.19 A
DHOX-21	47.00 ± 1.37 B	4.7 ± 2.6 B	6.74 ± 0.02 BC	73.75 ± 5.31 A	89.33 ± 1.27 A
DHOX-51	45.13 ± 2.70 B	4.3 ± 1.6 B	6.65 ± 0.07 C	74 ± 6.67 A	92.65 ± 1.84 A
DHOX-61	43.00 ± 1.62 B	4.3 ± 1.8 B	6.75 ± 0.04 BC	74.5 ± 5.41 A	90.05 ± 2.60 A

*^a^Number of rice false smut balls on the inoculated spike. ^b^Data from at least four replicates were analyzed with the protected Fisher’s least significant difference (LSD) test. Different letters mark statistically significant differences (P ≤ 0.01).*

### UvHOX2 Is Not Critical for Mycelia Growth and Conidium Germination

In addition, mycelium growth rate and conidium germination rate of the *UvHox2* deletion mutants was detected on YT media. The mycelium growth of *UvHox2* deletion mutants were slightly reduced comparing to wild-type strains. And no significant differences in the germination of conidia were observed between the *UvHox2* deletion mutant DHOX-61 and the wild-type strain P-1 (Table 2). This indicates that UvHOX2 is not critical in the regulation of mycelium growth and conidium germination in *U. virens*.

### *UvHox2* Deletion Mutants Exhibited Increased Sensitivity to Oxidative, Osmotic, and Cell Wall Stresses

**FIGURE 6:**
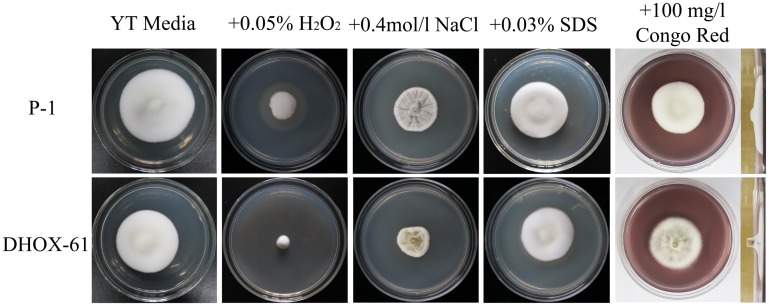
Growth of the *UvHOX2* deletion mutant in the presence of different biotic stresses. The wild-type strain P-1, *UvHOX2* deletion mutant DHOX-61 were cultured on YT medium or amended with 0.05% H_2_O_2_, 0.4 mol/l NaCl, 0.03% SDS and 100 mg/l congo red. Photographs were taken after incubation at 28°C for 15 days.

When cultured on YT media amended with 0.05% H_2_O_2_ and 0.4 mol/l NaCl, the colony diameter of the *UvHox2* deletion mutant DHOX-61 was significantly smaller than that of the wild-type strain P-1. When cultured on YT amended with 0.03% SDS and 100 mg/l congo red, the colony diameter of DHOX-61 was equivalent to that of P-1. However, the colony of DHOX-61 cultured on 100 mg/l congo red had shrinks and less aerial mycelia than P-1 (Table 3 and Figure 6). These findings showed that the *UvHox2* deletion mutants were more sensitive to oxidative, osmotic and cell wall stresses than the wild-type strains. It suggests that UvHOX2 is also involved in responses to oxidative, osmotic, and cell wall stresses.

**Table 3:** Responses of mycelium growth to abiotic stresses of *UvHOX2* deletion mutants.

Strain	Inhibition rate of mycelium growth (%)			
	H_2_O_2_	NaCl	SDS	Congo red
P-1	67.52 ± 3.55 B^a^	48.78 ± 2.14 C	35.37 ± 1.43 A	36.43 ± 3.76 A
DHOX-17	79.33 ± 1.25 A	55.87 ± 2.60 AB	24.80 ± 2.91 AB	27.04 ± 3.16 AB
DHOX-21	79.26 ± 1.93 A	60.64 ± 2.63 AB	32.98 ± 5.97 A	33.78 ± 4.91 AB
DHOX-51	76.18 ± 3.02 A	62.66 ± 2.14 A	28.82 ± 5.93 AB	28.26 ± 5.45 AB
DHOX-61	77.91 ± 1.87 A	54.94 ± 3.93 BC	23.26 ± 1.83 B	23.72 ± 3.79 B

*^a^Protected Fisher’s least significant difference (LSD) test was used for statistical analysis. Different letters mark statistically significant differences (P ≤ 0.01).*

### Subcellular Location and Expression Patterns of UvHOX2

**FIGURE 7:**
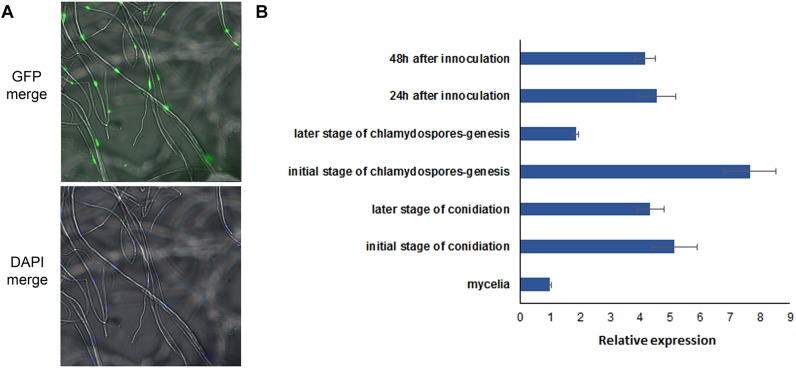
Location and expression pattern of UvHOX2 in *U. virens*. **(A)** UvHOX2-eGFP was activated and located in nuclei of mycelia. 4′,6-diamidino-2-phenylindole (DAPI) was used to stain nuclei in cells. **(B)** Expression pattern of *UvHOX2* was determined by qRT-PCR. House-keeping gene elongation factor 1-α (*EF1-α*) was employed as a reference gene.

The UvHOX2-eGFP construct was transformed into wild-type strain P-1. The mutant HOX-GFP-2 was picked from a batch of transformants for its strong signal of the over-expressed eGFP fusion protein. The eGFP signal in the HOX-GFP-2 mycelia located in the nuclei (Figure 7A). 4′,6-diamidino-2-phenylindole (DAPI) was used to dye the mycelia and show the location of the nuclei in the cells.

The expression levels of *UvHox2* at the stages of mycelium growth, conidium generation, and chlamydospore formation were determined by qRT-PCR. The results showed that the expression of *UvHox2* was the highest during the early stage of chlamydospore formation and decreased at the later stage of chlamydospore formation. Meanwhile, the expression of *UvHox2* was also high at the sporulating stage and the early stage of infection on rice. In contrast, the expression of *UvHox2* was low during vegetative growth. (Figure 7B).

### The Global Transcription Pattern of *UvHox2* Deletion Mutant Differs From That of the Wild Type at the Early Stage of Chlamydospore Development

**FIGURE 8:**
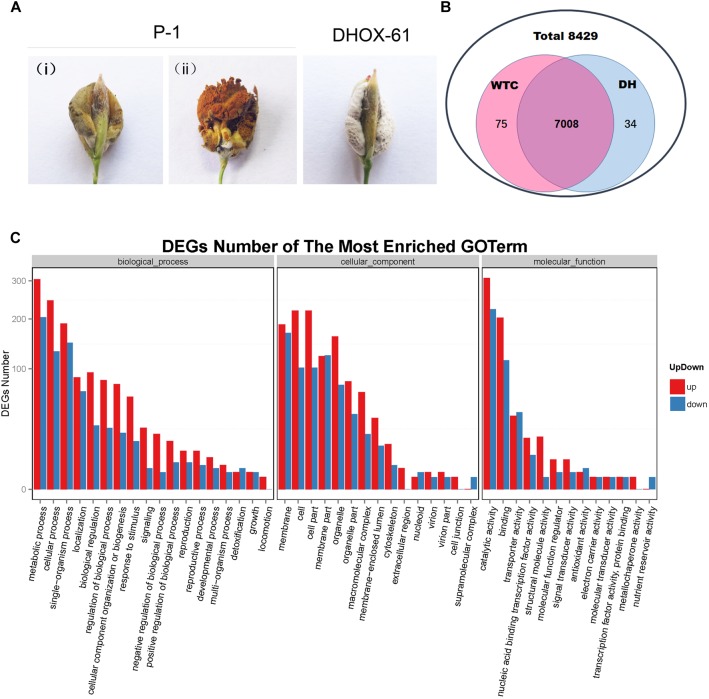
Comparative transcriptional analysis of genes regulated by UvHOX2 during chlamydospore development. **(A)** False smut ball samples were collected to perform RNA-seq and qRT-PCR assay. P-1 (wild-type strain of *U. virens*): (i) false smut balls at the initial stage of chlamydospore generation (WTC sample); (ii) false smut balls at the later stage of chlamydospore generation; DHOX-61 (*UvHox2* deletion mutant of *U. virens*): false smut balls of DHOX-61 at the initial stage of chlamydospore formation. **(B)** Mapping and assembly statistics for WTC and DH samples. **(C)** Gene Ontology (GO) term of differentially expressed genes in DH vs. WTC. The most enriched GO terms were biological processes, cellular components, and molecular function.

To identify putative regulated targets of the homeobox TF UvHOX2 during the formation of the chlamydospore generation structure, we compared the global gene transcription patterns in the *UvHox2* deletion mutant DHOX-61 with that in the wild-type strain P-1 by RNA-seq analysis. We inoculate P-1 (WTC) and DHOX-61 (DH) on rice as described above and collected rice false balls after 21 days. For false smut ball generated by P-1 (WTC), the membrane-like structure covering the false smut balls were intact then, and chlamydospore formation was at the early stage (Figure 8A).

The raw data of the RNA-seq was deposited in GenBank (accession number: SUB4385058). There was high Pearson correlation among duplicates. We found that the global transcription patterns of DH and WTC at the early stage of chlamydospore formation differed considerably. In a total of 8429 genes identified in *U. virens* previously (Zhang et al., 2014), 75 genes excluding *UvHox2* were only expressed in WTC, 34 genes were barely expressed in DH, and 7008 overlapping genes were expressed in both WTC and DH (Figure 8B and Supplementary Table S2). Furthermore, we identified 1877 genes that have differential expression by at least twofold between WTC and DH, including 1185 genes being up-regulated and 692 genes being down-regulated in DH vs. WTC. These genes constituted approximately one-half of the differentially expressed genes, and they could be assigned to three major functional groups: biological process, cellular component, and molecular function (Figure 8C and Supplementary Table S2). In the biological process group, the top three subgroups of differentially regulated genes in DH vs. WTC were “metabolic process,” “cellular process,” and “single-organism process.” In the cellular component group, the top three subgroups of differentially expressed genes in DH vs. WTC were “membrane,” “cell,” and “cell part.” In the molecular function group, the top three subgroups of differentially expressed genes in DH vs. WTC were “catalytic activity,” “binding,” and “transporter activity.”

To validate the RNA-seq data, quantitative real-time polymerase chain reaction (qRT-PCR) was performed to confirm the differential expression of six selected genes. The qRT-PCR data for these genes were consistent with those obtained from RNA-seq (Supplementary Figure S2).

### Genes Involved in Signal-Transduction Pathway

Several differentially expressed genes were detected in WTC, which were regarded as components in signal recognition and transduction system (Table 4). We also found that 43 genes encoding TFs were up-regulated during chlamydospore formation (Supplementary Table S2). And 19 of these genes were up-regulated beyond four folds (Table 4).

**Table 4:** A portion of genes that may under the control *UvHOX2.*

Genes (accession numbers)	Biological function	Up-/down-regulated^a^
**Genes involved in signal transduction**		
KDB16312	G-protein coupled receptors	Up
KDB11861	G-protein coupled receptors	Up
KDB16287	G-protein coupled receptors	Up
KDB11050	G-protein coupled receptors	Down
KDB19029	Gtr1/RagA G protein	Up
KDB19030	Gtr1/RagA G protein	Up
KDB14836	Rho GTPase protein	Up
KDB11145	Serine/Threonine-protein phosphatase 6 subunit	Up
KDB12061	Serine/Threonine-protein kinase IO1	Up
KDB17370	Protein kinase	Up
KDB17112	Ethanolamine kinase	Up
KDB18374	TBC domain containing protein	Up
KDB15779	C_2_H_2_-type transcription factors	Up
KDB12683	C_2_H_2_-type transcription factors	Up
KDB12684	C_2_H_2_-type transcription factors	Up
KDB17197	C_2_H_2_-type transcription factors	Up
KDB17551	C_2_H_2_-type transcription factors	Up
KDB11104	bZIP transcription factors	Up
KDB14749	bZIP transcription factors	Up
KDB17948	C6 transcription factors	Up
KDB18664	C6 transcription factors	Up
KDB11753	Zinc finger protein odd-paired-like protein	Up
KDB13074	Homeobox transcription factor	Up
KDB14479	ACE1	Up
KDB17109	bHLH family transcription factor	Up
KDB18696	Transcription factor	Up
KDB12822	Transcription factor	Up
KDB15421	Transcription factor	Up
KDB11243	Transcription factor	Up
**Genes involved in autophagy and ubiquitination**		
KDB17685	Autophagy relative protein 3	Up
KDB14954	Autophagy relative protein 5	Up
KDB12188	Autophagy relative protein 7	Up
KDB15378	Autophagy relative protein 12	Up
KDB13656	Autophagy relative protein 26	Up
KDB16605	Autophagy relative protein 22	Up
KDB14455	Snf2 family ubiquitin-protein ligase (Kwon et al., 2013)	Up
KDB14890	Cullin 3 (McEwan and Dikic, 2014; Casanova et al., 2015)	Up
KDB17984	Target of rapamycin complex 2 (TORC2) subunit (Inoue and Klionsky, 2010)	Up
**Proteins involved in osmotic response and cell membrane integrity**		
KDB18369	Sensor histidine kinase TcsA	Up
KDB18143	Sensor histidine kinase TcsA	Up
KDB18379	NIK1	Up
KDB11585	SLN1	Up
KDB12998	Phosphorelay intermediate protein YPD1	Up
KDB11673	Transcription regulatory protein TUP1	Up
KDB12278	MFS transporter	Up
KDB17072	MFS multidrug transporter	Up
KDB12738	Calcium ion binding protein	Up
KDB18607	ABC transporter	Up
KDB16091	Mitochondrial calcium uniporter protein	Up
KDB11501	Phosphatase beta protein	Up
KDB16963	Plasma membrane channel protein Aqy1	Up
KDB17556	Glycoside hydrolase family 3 protein	Up

*^a^Up- or down-regulated genes at the early stage of chlamydospore formation of wild-type stain P-1 comparing to *UvHOX2* deletion mutant DHOX-61.*

### Genes Involved in Cell Wall Synthesis

Several differentially expressed genes were found to be closely linked to cell wall integrity. A gene encoding chitin deacetylase (KDB11455) were specially expressed in WTC but not in DH. Meanwhile, a chitin synthase (KDB11224) gene was up-regulated in WTC compared to DH. Chlamydospores of *U. virens* have thick cell walls. Chitin is an important component in cell wall, and fungi might mask chitin by deacetylating it into chitosan (Cord-Landwehr et al., 2016). These chitin synthases and deacetylase may play a key role in the thin cell wall synthesis in chlamydospores.

### Genes Involved in Ubiquitination and Autophagy

Autophagy is a kind of intracellular recycling system that degrade cytoplasmic materials in lysosome/vacuole during development and in response to cell stresses in eukaryotic cells (Liu et al., 2017). During chlamydospore formation, a lot of cytoplasmic materials may be degraded and reutilized. Here we identified three genes involved in autophagy that were differentially expressed in DH vs. WTC (Table 4).

### Genes Involved in Osmotic Response and Cell Membrane Integrity

We found ten osmotic stress responsive genes were differentially expressed in DH vs. WTC. Nine of these genes encoding components for osmolarity two-component response system were up-regulated in DH vs. WTC. Meanwhile, several genes encoding cell membrane components were found to be up-regulated in WTC (Table 4). Most of them are transporter genes, which suggested that *U. virens* cells need to exchange substances with the environment more frequently during chlamydospore formation.

### Generation of Chlamydospores and Conidia Might Share a BrlA-AbaA-WetA Regulatory Pathway

**FIGURE 9:**
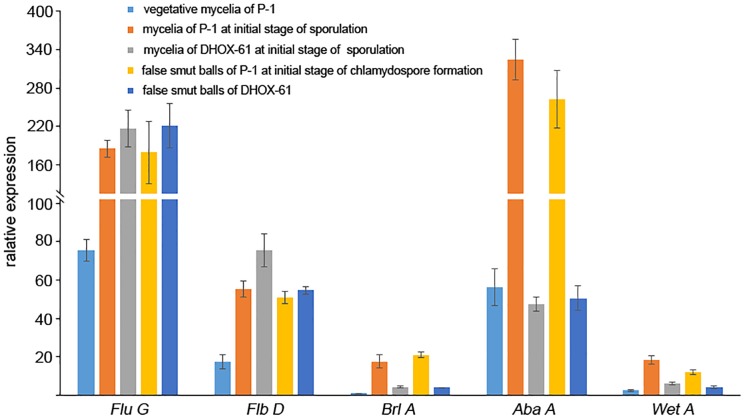
Expression of regulatory factors during chlamydospore and conidium formation in *U. virens* wild-type strain P-1 and *UvHOX2* deletion mutant DHOX-61. The relative expression level of *Flu D*, *Flb D*, *Brl A*, *Aba A*, and *Wet A* at vegetative mycelia of P-1, mycelia of P-1 at initial stage of sporulation, mycelia of DHOX-61 at initial stage of sporulation stage, false smut balls of P-1 at initial stage of chlamydospores formation of *U. virens*, and false smut balls of DHOX-61.

In *Aspergillus nidulans*, Myb-like DNA-binding protein FlbD is required for early conidiophore development (Wieser and Adams, 1995; Dong et al., 2015; Matheis et al., 2017). FluG regulates FlbD via repressing SfgA, a negative regulator of FlbD. FlbD delivers signals to the down-stream regulatory component FlbB to activate conidiogenesis regulatory cascade BrlA-AbaA-WetA (Wu et al., 2018). In *U. virens*, we found that homologs of *FluG* (KDB12888), *FlbD* (KDB18803), *BrlA* (KDB11753), *AbaA* (KDB11305), and *WetA* (KDB15008) were expressed at a higher level at the initial stage of chlamydospore and conidim formation than that at the vegetative growth stage. Homologs of *FluG* and *FlbD* were up-regulated, while homologs of *BrlA*, *AbaA*, and *WetA* were down-regulated in both initial sporulation mycelia and false smut ball at the initial stage of chlamydospore formation in DH compared to WTC (Figure 9). This suggested that the generation of chlamydospores and conidia may share the BrlA-AbaA-WetA regulatory pathway, and BrlA-AbaA-WetA signal cascade was downstream the UvHOX2 regulation.

## Discussion

Chlamydospores are a type of asexual spores that allow fungi to survive unfavorable conditions. These thick-wall spores play important roles in epidemic of pathogenic fungi as asexual resting spores and/or infection resources. Famous chlamydospore producing plant pathogenic fungi include *U. virens* (Zhang et al., 2014), *Fusarium oxysporum* (Klein et al., 2011), *F. sporotrichioides, and F. graminearum* (Goh et al., 2009). Some well-studied fungal biocontrol agents in livestocks, for example, *Trichoderma* spp. (Li et al., 2005), *Metarhizium anisopliae* (Ment et al., 2010), *Pochonia chlamydosporia* (Wang et al., 2005), and *Clonostachys rosea* (Ahmed et al., 2014), also produce chlamydospores. The development of chlamydospore is generally controlled by regulatory networks stimulated by the environment. Here, we report a homeobox TF UvHOX2 that is essential for chlamydospore formation and also contributes to pathogenicity in *U. virens.* Additionally, we identified a group of genes that may participate in the downstream regulatory network of UvHOX2 during chlamydospore formation.

Homeobox domain-containing proteins play a critical role in the regulatory network of fungal development and pathogenicity as downstream elements in plant pathogenic fungi, but the number of homeobox genes varies (Kim et al., 2009). Since the homolog of the transcription motif of STE12 in fungi is distinct from the typical homeodomain, we did not take account of the homolog of *STE12* (KDB11415) as a homeobox gene. As a result, we identified seven genes encoding homeobox containing proteins in *U. virens*. Two of them contain multiple DNA-binding motifs besides homeobox motif. Only one homeobox DNA-binding motif was identified in the N-terminus of UvHOX2. In ascomycetous fungi, orthologs of UvHOX2 may play conserved roles in the development of specific structures during sporulation. In *Verticillium dahliae*, deletion of *vhb1* (homolog of *UvHox2*) reduced sporulation rates in liquid medium (Sarmiento-Villamil et al., 2018). In *Fusarium* species, the deletion mutants failed to form conventional phialides and had obstructions in generating microconidia, but it could still produce macroconidia which was formed from hyphae through a budding-like mechanism (Zheng et al., 2012). In plant pathogen *Magnaporthe oryzea*, the *Mohtf1* gene (homolog of *UvHox2*) is essential for conidiation but not for hypha growth and pathogenicity. The *Mohtf1* deletion mutants generated more conidiophores, which failed to develop into sterigmata-like structures (Kim et al., 2009; Liu et al., 2010). Accordingly, deletion of the homolog of *UvHox2* caused obstructions in conidiophore-genesis and completely abolished the generation structure of chlamydospores.

Conidia and chlamydospores are asexual spores produced by *U. virens*. In *A. nidulans*, regulatory factors, BrlA, AbaA, and WetA, were considered as cell developmental regulators that were critical for the development of conidiophore and phialide, as well as spore maturation (Cary et al., 2017; Wu et al., 2018). Here, we provided a clue that BrlA-AbaA-WetA cascade may also participate in the regulation of chlamydospore formation in *U. virens*. Deletion of *UvHox2* reduced the expression of *BrlA*, *AbaA*, and *WetA*, but the deletion did not affect the upstream regulatory factors FluG and FlbD. This suggested that UvHOX2 and FlbD regulatory pathways could coordinate to regulate the downstream BrlA-AbaA-WetA cascade during sporulation and chlamydospore formation in *U. virens*. Although deletion of *UvHox2* did not completely block the *BrlA*-*AbaA*-*WetA* signal cascade, the *UvHox2* deletion mutant lost the ability to form special structures for chlamydospore generation. UvHOX2 must control other regulatory pathways, which are critical for generating special structures during chlamydospore formation. Moreover, because conidia generated by *UvHox2* deletion mutant could convert into chlamydospore as wild-type strains do, UvHOX2 may not be critical for maturation of chlamydospores in *U. virens*.

The two-component signaling proteins responsive to osmotic stress play an important role in cell tolerance under adverse environmental conditions. Our results revealed that Sln1 and Skn7, which were differentially expressed in DH vs. WTC, may function downstream of the UvHOX2 signaling pathway. Although the histidine kinase protein Sln1 and TF Skn7 play important roles in responses to osmotic, oxidative, and cell wall stresses (Zhang et al., 2010; Tang et al., 2017), their exact functions vary among fungi. The infection process of *U. virens* is unique compared to most phytopathogenic fungal pathogens (do not penetrate or destroy the host cells after infection) and the morphology of this fungi vary (Tang et al., 2013; Fan et al., 2014; Zhang et al., 2014; Yu J.J. et al., 2015; Song et al., 2016). The exact roles of these responsive proteins in cell development and pathogenesis need to be uncovered in the future.

The low frequency of homologous gene replacement in *U. virens* had limited its genetic study at the molecular level in the past years. Recently, a successful gene-deletion system based on CRISPR-Cas9 has been developed by Liang et al. (2018). It makes it convenient and efficient to perform gene deletion in *U. virens*. Using the optimized CRISPR-Cas9 cassettes, we developed a gene deletion system based on ATMT transformation and CRISPR-Cas9. This gene-deletion system shortens culture-selection-period by 7–10 days and provides a valuable tool for molecular genetic study in *U. virens*.

## Author Contributions

YL and JY conceived and designed the experiments. JY, MYu, ZQ, MYo, RZ, and XY performed the experiments. JY, TS, HC, XP, and YD analyzed the data. YL contributed to reagents, materials, and analysis tools. JY and YL wrote the manuscript.

## Conflict of Interest Statement

The authors declare that the research was conducted in the absence of any commercial or financial relationships that could be construed as a potential conflict of interest.
