# Supplementary material for: A Homeobox Transcription Factor UvHOX2 Regulates Chlamydospore Formation, Conidiogenesis, and Pathogenicity in Ustilaginoidea virens
Source: Front Microbiol. 2019 Jun 20;10:1071. doi: 10.3389/fmicb.2019.01071 (PMC6596325; doi:10.3389/fmicb.2019.01071)
Supplement: Supplementary file 1 [file Table_1.docx]

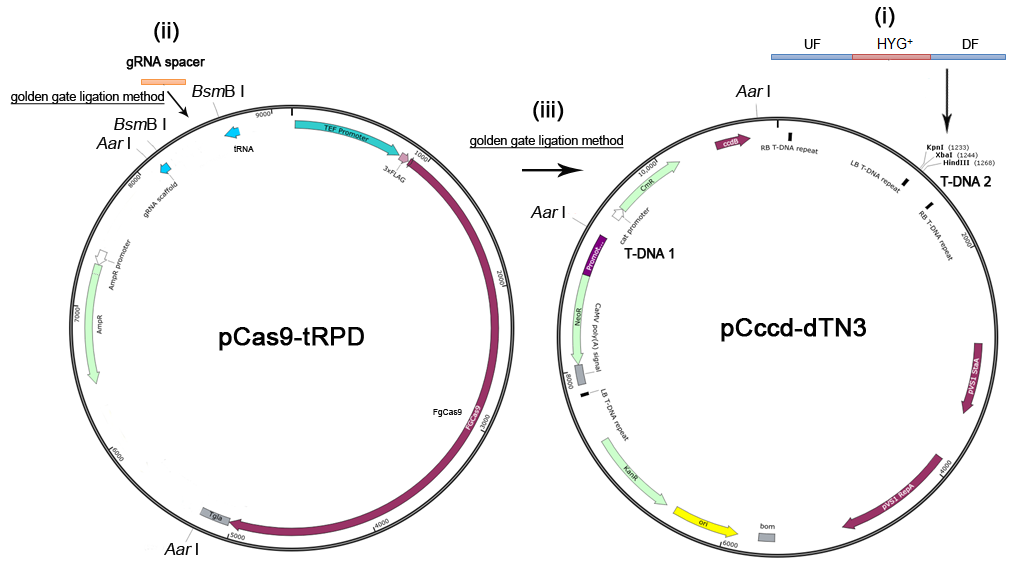


**Supplementary FIGURE S1 │Construction procedure of binary vector for targeted gene replacement based on CRISPR/Cas9.** Construction of receptor binary vector pCccd-dTN3 with two T-DNA regions. The *ccdB* cassette, having *Aar* I restriction sites were directed into two ends, was amplified from pType IIs (Invitrogen) with primer pair P1-P2 (Supplementary TABLE S1) and cloned into large fragment of pCambia1300 cut by *Xho* I and *Bst* XI to generate pCccd. The mCherry cassette was amplified from pmCherry-hph with primer pair P3-P4 (Supplementary TABLE S1), the sequences of Left-border (LB) and Right-border (RB) were introduced into ends of fragment, the mCherry cassette was subsequently ligated to *Eco*R I-*Kpn* I large fragment of pCccd to construct pCccd-dT. This pCccd-dT contains two T-DNA region (T-DNA 1 and T-DNA 2) separated by 1.1-kb mCherry cassette. Then, a neomycin resistant cassette (NEO^+^), amplified from plasmid pkntg with primer pair P5-P6 (Supplementary TABLE S1), was ligated to pCccd-dT cut by *Xho* I to generate pCccd-dTN3. We used ClonExpress Ultra One Step Cloning Kit (Vazyme) in plasmid construction in this study. Construction of pCas9-tRPD. We used [Mut Express II Fast Mutagenesis Kit V2](http://www.vazyme.com/products_detail/productId=148.html) (Vazyme) and primer pairs P15-P16 and P17-P18 (Supplementary TABLE S1) to insert two *Aar* I restriction sites into flanks of Cas9-gRNA cassettes of pCas9-tRp-gRNA (Liang et al. 2018), respectively, to generate pCas9-tRPD. The *UvHOX2* deletion binary vector was constructed as following: **(i)** Construction of binary vector with targeted gene replacement cassette. A 1.1 kb upstream flank of UvHox2 coding region (UF) and a 1kb downstream flank of UvHox2 coding region (DF) were amplified with primer pairs P7-P8 and P9-P10 (Supplementary TABLE S1), respectively. The 1.4kb hygromycin resistant cassette (HYG^+^) under the control of promoter TrpC (PtrpC) was amplified with primer pair P11-P12 (Supplementary TABLE S1). Double-joint PCR method ([Yu et al., 2004](#_ENREF_50)) was employed to ligate fragments in the order of UF-HYG^+^-DF. A pair of nest primers P13-P14 (Supplementary TABLE S1) was used in the last round of PCR. The UF-HYG^+^-DF cassette was subcloned into the *Kpn* I-*Hin*d III restriction sites (in T-DNA 2 region) of pCccd-dTN3 to form pCccd-dTN3-HX2. *Escherichia coli* DB3.1 competent cells (TransGene Biotech) resistant to ccdB toxic protein were used in construction of vector containing ccdB cassette. **(ii)** Construction of CRISPR/Cas9-gRNA donator plasmid. The 20-bp sense and anti-sense oligonucleotides of gRNA spacers SP1S-SP1AS and SP2S-SP2AS (Supplementary TABLE S1) were designed using online tool (<http://grna.ctegd.uga.edu/>). The sgRNA candidates highly rated by the software were further filtered via off-target analysis as described by Liang et al. (2018). These gRNA spacers were synthesized with adaptors at the 5’ end (sense: 5’-ACCT-3’ and anti-sense: 5’-AAAC-3’), inserted into *Bsm*B I sites of pCas9-tRPD to generate pCas9-tRPDI and pCas9-tRPDII via Golden gate method, respectively ([Arazoe et al., 2015](#_ENREF_4);[Liang et al., 2018](#_ENREF_28)). **(iii)** Construction of gene deletion vector with CRISPR/Cas9. The CRISPR/Cas9-gRNA cassette of donator vector pCas9-tRPDI or pCas9-tRPDII was used to replace the ccdB cassette in pCccd-dTN3-HX2 to generate pdTN3-HX2-Cas9I and pdTN3-HX2-Cas9II via Golden gate method ([Engler and Marillonnet, 2014](#_ENREF_13);[Arazoe et al., 2015](#_ENREF_4)). The protocol was modified slightly as follows: 0.02 pmol pCccd-dTN3-HX2, 0.06 pmol pCas9-tRPD1 or pCas9-tRPD2, 1.6 μl 10×T4 DNA ligase buffer, 0.3 µl 50×oligonucleotide (0.025 mM), 0.8 μl *Aar* I (Thermo), T4 DNA ligase (NEB), and ddH_2_O were mixed with a total volume of 16 μl. Reaction was performed for 6 cycles (37°C for 5 min, 25°C for 10min). Subsequently, 1 μl *Aar* I, 2 μl 10×digestion buffer, 0.1 μl 50×oligonucleotide (0.025 mM) and ddH_2_O was added to the reaction to a total volume of 20 μl. The digestion reaction was performed at 37°C for 1h and thentransformed into *E. coli* DH5α competent cells.

**References**

Arazoe, T., Miyoshi, K., Yamato, T., Ogawa, T., Ohsato, S., Arie, T., and Kuwata, S. (2015). Tailor‐made CRISPR/Cas system for highly efficient targeted gene replacement in the rice blast fungus. *Biotechnol and Bioeng* 112**,** 2543-2549.

Engler, C., and Marillonnet, S. (2014). "Golden gate cloning," in *DNA cloning and assembly methods*. Springer), 119-131.

Yu, J.-H., Hamari, Z., Han, K.-H., Seo, J.-A., Reyes-Domínguez, Y., and Scazzocchio, C. (2004). Double-joint PCR: a PCR-based molecular tool for gene manipulations in filamentous fungi. *Fungal Genet and Biol* 41**,** 973-981.


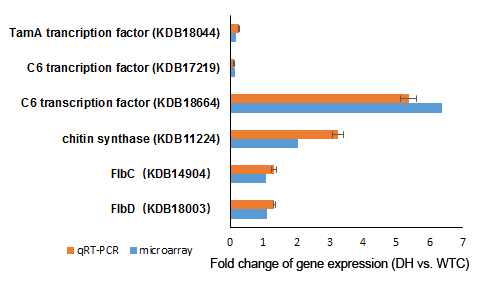


**Supplementary FIGURE S2**│ Validation of the differentially expressed genes in *UvHOX2* deletion mutant (DH) vs. wild-type strain (WTC) of *U. virens* . The gene expression fold change of KDB18044, KDB17219, KDB18664, KDB11224, KDB14904 and KDB18003 were calculated by base-mean value of microarray assay and qRT-PCR.

| **Primers** | **Sequences (5’ to 3’)** |
| --- | --- |
| P1 | CATTATTATGGAGAAACTCGAGCTGGGTTGGCAGGTGCTGGAGA |
| P2 | GAGCAGCTTGCCAACATGGTGGATGCGCATGCAGGTGATGCGGAG |
| P3 | GACCATGATTACGAATTCGAGTGACAGGATATATTGGCGGGTAAACTCACTTGTACAGCTCGTCCATG |
| P4 | AGAGGATCCCCGGGTACCGAGGTTTACACCACAATATATCCTGCCAACAGAAGATGATATTGAAG |
| P5 | CCAGCACCTGCCAACCCAGCTCGAGAGGGCGAATTGGGTACTC |
| P6 | ACACATTATTATGGAGAAACTCAGAAGAACTCGTCAAGAAGGCGA |
| P7 | AAACCTCGGTACCCGGGGATCCTCTAGACCCATCTTGGCACCCTACAAG |
| P8 | GCTCCTTCAATATCATCTTCTGTCAAGACGGTCCCACAGCTAC |
| P9 | CGAGGGCAAAGGAATAGAGTAAGTGTTGTGGACCGAGTGGT |
| P10 | CGAGGGCAAAGGAATAGAGTAACGACATGGCATGTACACCGT |
| P11 | ACAGAAGATGATATTGAAGGAGC |
| P12 | TACTCTATTCCTTTGCCCTCG |
| P13 | AAACCTCGGTACCCGGGGATCCTCTAGAGCACCCAAGCACCCACGA |
| P14 | TTGTAAAACGACGGCCAGTGCCAAGCTTTGGGGTCAAGATGCCAGAGTG |
| P15 | TACACCTGCATGCATGCTCAGGGCGATGGCCCACTAC |
| P16 | ACCACCTGCCAGCCTGGCGAGCGCAGCGAGTCAGTGAG |
| P17 | CGCCAGGCTGGCAGGTGGTCGTTCGGCTGCGGCGAGCGGTATC |
| P18 | GAGCATGCATGCAGGTGTAGACGGTTTTTCGCCCTTTGACGT |
| P19 | GAAGACGCTTGCCGAGTCCA |
| P20 | GATTGTCGGCAGTCCGAAGA |
| P21 | GAAGTGCTTGACATTGGGGA |
| P22 | TCGTCCATCACAGTTTGCCA |
| P23 | ATCAGGTCGGAGACGCTGTC |
| P24 | CCCATCTTGGCACCCTACAAG |
| P25 | TGGCAAACTGTGATGGACGA |
| P26 | GGATGCGACGAACCTTGATC |
| P27 | GAGGAACCCAATCTTCAAAGATGGGACCATACTCTCACTCAC |
| P28 | TCCGCCCCCGCCACCTCCACTGTCAATTTGGTGGAGGCTCA |
| P29 | AGTGGAGGTGGCGGGGGCGGAATGGTGAGCAAGGGCGAGGAG |
| P30 | GGATTGATTGTTTAATTAAGTTACTTGTACAGCTCGTCCATG |
| SP1S | ACCTGGACGTTGACACGCCCGCAT |
| SP1AS | AAACATGCGGGCGTGTCAACGTCC |
| SP2S | ACCTGACTAGCCAGAGCGTTGAAA |
| SP2AS | AAACTTTCAACGCTCTGGCTAGTC |

**Supplementary TABLE S1│** Primers used in this study.

**Supplementary TABLE S2│** Differentially expressed genes in DH vs. WTC. (see in excel file)
